# Clinical Effectiveness of Various Surgical Reconstruction Modalities for Acute ACJ Separation: Protocol for a Systematic Review and Meta-Analysis

**DOI:** 10.29337/ijsp.172

**Published:** 2022-03-03

**Authors:** Alexander W. Hartland, Sandeep Krishan Nayar, Kar Hao Teoh, Mustafa S. Rashid

**Affiliations:** 1 Northampton General Hospital, Cliftonville, Northampton, NN1 5BD, UK; 2 Royal London Hospital, Whitechapel Rd, London, E1 1FR, UK; 3 Trauma and Orthopaedics, Princess Alexandra Hospital, Hamstel Road, Harlow, Essex, CM20 1QX, UK; 4 Nuffield Department of Orthopaedics, Rheumatology, and Musculoskeletal Sciences, Windmill Road, Oxford, OX3 7LD, UK

**Keywords:** acromioclavicular, dislocation, separation, acute, Rockwood, coracoclavicular

## Abstract

**Introduction:**

Acute acromioclavicular joint separation is a common injury to the shoulder. Various surgical reconstruction methods exist when operative management is required, but the optimal procedure is not known. The aim of this systematic review and meta-analysis is to review the literature to assess the clinical effectiveness of various surgical reconstruction modalities used for acute ACJ separation.

**Methods:**

The study protocol was designed and registered prospectively on PROSPERO (International prospective register for systematic reviews). Literature search will include MEDLINE, EMBASE, PsycINFO, and The Cochrane Library electronic databases. Randomised controlled trials (RCTs) evaluating surgical procedures for acute acromioclavicular joint (ACJ) separation will be included. Our primary outcome is any functional patient-reported outcome measure related to the shoulder. Secondary outcomes may include radiological measurements, objective measurements of strength testing, range of motion, other patient-reported outcome measures not specific to the shoulder such as the Visual-Analog Scale (VAS) for pain, timelines for return to sport or work, and rate of complications. Risk of bias will be assessed within each study using The Cochrane Risk of Bias Tool 2.0 and the Jadad score. Inconsistency and bias across included studies will be assessed statistically. Comparable outcome data will be pooled and analysed quantitatively or qualitatively as appropriate.

**Ethics and dissemination:**

This study did not require ethical clearance. We plan to publish this systematic review and meta-analysis in a peer-reviewed journal and present the results at various national and international conferences.

**Highlights:**

- There is currently variation in surgical synthetic ligament reconstruction techniques for acute acromioclavicular separation, with no clear consensus established.
- This systematic review evaluates the clinical effectiveness of various surgical reconstruction modalities used for acute ACJ separation.
- Our primary outcome is any functional patient-reported outcome measure related to the shoulder.

## 1. Introduction

Acromioclavicular joint (ACJ) separation is a common shoulder injury, particularly amongst men and athletes. It is a source of pain and instability, due to traumatic injury to the ACJ. It is associated with disruption of the acromioclavicular (AC) ligaments and/or the coracoclavicular (CC) ligaments. Diagnosis can be made through clinical examination and radiological assessment. Management is dependent on the severity of the injury, degree of horizontal plane instability, pain, and patients’ expectations. Options include both conservative and surgical strategies. The Rockwood classification is frequently referred to as an aid in treatment decisions [1]. This classification system categorises injury into types I–VI based on AC and CC ligament injury, clinical examination, radiographic evaluation, and reducibility. It is generally accepted that types I and II can be treated conservatively with short term sling immobilisation and early appropriate rehabilitation. Types IV–VI will often require surgical management to restore stability. The treatment for type III injury remains controversial, with many patients often returning to pre-injury function following a period of conservative management. Whilst others will require delayed reconstruction to restore function and alleviate pain.

When surgical management is indicated, the optimal surgical procedure to provide maximal clinical benefit remains unclear. Various surgical procedures have been described. These include artificial ligament reconstruction; the use of an autograft/allograft; temporary screw fixation via open reduction and internal fixation; and open reduction of the separation using a hook plate and screws. Screw fixation from the clavicle to the coracoid and hook plate techniques require removal after a period of several months. Synthetic ligament reconstruction techniques often utilise multifilament braided ultra-high molecular weight polyethylene sutures/tape either wrapped around the coracoid/clavicle or passed via bone tunnels and secured. Some utilise endobutton(s) to provide suspensory fixation on the clavicle and/or the coracoid. Many variations exist, some utilising multiple tails, supplemented with allograft/autograft tissue. The purpose of this meta-analysis and systematic review is to assess the clinical effectiveness of various surgical reconstruction modalities used for acute ACJ separation.

## 2. Objectives

1. Which surgical technique offers the most superior clinical improvement in acute ACJ dislocations?
2. Which surgical technique performed for acute ACJ dislocation yields the most favourable risk profile?

## 3. Methods

This study protocol was registered prospectively on the PROSPERO (International prospective register for systematic reviews) database (Ref: CRD42021291349). It is reported according to the Preferred Reporting Items for Systematic reviews and Meta-Analyses Protocol (PRISMA-P) [2,3].

### 3.1 Eligibility criteria

#### 3.1.1 Study design

Only randomised controlled trials will be included. All other trial designs will be excluded.

#### 3.1.2 Participants

Studies with human patients of any age undergoing any type of surgery for acute acromioclavicular joint separation will be included. This may include open and arthroscopic techniques.

#### 3.1.3 Intervention and comparators

The intervention of interest is surgical synthetic ligament reconstruction techniques. The comparators will be any alternative surgical procedures used for acute ACJ separation which may include, ligament autograft/allograft reconstruction, modified Weaver Dunn techniques, and Hook plate techniques.

#### 3.1.4 Outcomes

The primary outcome of interest will be any functional patient-reported outcome measures related to the shoulder. This may include the Oxford Shoulder Score (OSS), the Constant-Murley Score (CMS), the American Shoulder and Elbow Surgeons Shoulder Score (ASES), and the Disabilities of the Arm, Shoulder, and Hand Score (DASH).

Secondary outcomes may include radiological measurements, objective measurements of strength testing, range of motion, other patient-reported outcome measures not specific to the shoulder such as the Visual Analog Scale (VAS) for pain, time for return to sport and/or work, and rate of complications.

#### 3.1.5 Timing

No restrictions placed on the timing of the study.

#### 3.1.6 Setting

No restrictions placed on the setting of the study.

#### 3.1.7 Language

No restrictions placed on the language of the study. Any studies requiring translation into English will be included in the appendix.

### 3.2 Information sources

Bibliographic databases searched included; MEDLINE, EMBASE, PsycINFO and The Cochrane Library.

#### 3.2.1 Search strategy

To increase sensitivity and heighten precision, the Cochrane group randomised controlled trial filters were used in the search strategy for each database [4]. An example of the search terms utilised are included in the appendix. References from published systematic reviews investigating the same or similar topic were also manually searched for relevant studies. No ongoing or recently completed systematic reviews on this topic were found within the PROSPERO database.

### 3.3 Study records

#### 3.3.1 Data management

All literature search results will be combined and collected in Endnote X9 (Clarivate Analytics). Duplicate articles will be removed. Two independent reviewers will screen titles and abstracts, with consensus sought prior to full text review. Full text review of all articles meeting the eligibility criteria will then determine final inclusion. If any dissonance exists that cannot be resolved, a third reviewer will review to determine inclusion.

#### 3.3.2 Data collection process

Data extraction will involve two independent reviewers. One reviewer will extract the required data using a standardised proforma. A second reviewer will then check the extracted data for any inaccuracies. Any differences found during the data extraction process will be resolved by discussion and the involvement of a third reviewer as needed. Where incomplete or missing data is encountered, attempts to contact authors of individual studies will be made. Microsoft Excel will be used for data capture and Review Manager (RevMan version 5.3) used for data management.

#### 3.3.3 Data items

Extracted data will include study design, patient cohort, study characteristics, surgical intervention, comparator surgical intervention, classification/grade of injury included, primary outcome measures, and any secondary outcome measures including complications and adverse events. Mean and standard deviations will be extracted for all outcome measures where possible. Items related to methodological design and reporting will be extracted to allow risk of bias assessment within studies.

### 3.4 Outcomes and prioritisation

#### 3.4.1 Primary outcome

The primary outcome of interest will be any functional patient-reported outcome measures related to the shoulder. This may include the Oxford Shoulder Score (OSS), the Constant-Murley Score (CMS), the American Shoulder and Elbow Surgeons Shoulder Score (ASES), and the Disabilities of the Arm, Shoulder, and Hand Score (DASH).

#### 3.4.2 Secondary outcomes

Secondary outcomes may include radiological measurements, objective measurements of strength testing, range of motion, other patient-reported outcome measures not specific to the shoulder such as the Visual Analog Scale (VAS) for pain, time to return to sport and/or work, and rate of complications.

### 3.5 Risk of bias of individual studies

The Cochrane Risk of Bias tool 2.0 will be utilised to assess for potential bias of individual studies [5]. This tool consists of 5 domains of bias, with each domain assigned a level of risk (high risk, low risk, or some concerns). Pre-determined signalling questions guide the interpretation of the risk of bias for each domain, and an overall risk of bias is subsequently generated. We will also use the Jadad scale, as a supplementary method for assessing bias [6]. The Jadad scale awards 0–5 points based on the application and method of randomisation used, the application and method of blinding used, and all patients involved in the trial being accounted for.

### 3.6 Data synthesis

#### 3.6.1 Quantitative synthesis

Where recorded outcomes are comparable across studies, data will be synthesised quantitatively, in the form of a forest plot. Assessment of heterogeneity between studies will be quantified using the chi-square test for heterogeneity and the *I*^2^ statistic. We will use a random effects model for analysis due to expected heterogeneity between studies. Continuous data will be summarised using standardised mean difference and inverse variance statistical analysis. Any dichotomous data presented will be measured for effect using odds ratios.

#### 3.6.2 Qualitative synthesis

Data will be reported descriptively if outcome measures reported are not comparable, the heterogeneity is too high, or the incidence of the event is too low for pooled statistical analysis.

#### 3.6.3 A priori subgroup analyses

From our inclusion criteria, subgroup analyses may be possible based on the method of surgical reconstruction used. This may include with or without allograft supplementation. Subgroup analyses based on the grade of ACJ injury, divided into high (Rockwood grade IV, V, and VI) and low grade (Rockwood I, II, and III) injuries will be performed. Subgroup analysis within high grade injuries (i.e. Rockwood grade IV vs V) will not be performed due to variation amongst studies with regards to accuracy of grading, variation in radiographic evaluation, and clinical significance on ACJ instability.

#### 3.6.4 Meta-bias

We will assess for publication bias using a funnel plot of included studies investigating our primary outcome. We will also assess for selective reporting within studies by reviewing available trial protocols or registrations to compare pre-defined outcomes with those analysed and reported in the published manuscript. The risk of bias within individual studies will be assessed as previously described. Bias across studies will be assessed using statistical analysis of heterogeneity, as a measure of inconsistency.

#### 3.6.5 Confidence in cumulative estimate

The strength of the body of evidence provided will be assessed using the Grading of Recommendations Assessment, Development and Evaluation (GRADE) approach [7,8,9]. Each outcome will be subsequently described as being of very low, low, moderate, or high certainty.

## Appendix

### Search terms for MEDLINE

1. Randomized controlled trial.pt.
2. Controlled clinical trial.pt.
3. Randomized.ab,ti.
4. Placebo.ab,ti.
5. Clinical trials as topic.me.
6. Randomly.ab,ti.
7. Trial.ti.
8. 1 OR 2 OR 3 OR 4 OR 5 OR 6 OR 7
9. Acromioclavicular.ab,ti.
10. Acromio*.ab,ti.
11. Clavic*.ab,ti.
12. ACJ.ab,ti.
13. Coracoclavicular.ab,ti.
14. Coraco-clavicular.ab,ti.
15. Conoid.ab,ti.
16. Trapezoid.ab,ti.
17. 9 OR 10 OR 11 OR 12 OR 13 OR 14 OR 15 OR 16
18. Separation.ab,ti.
19. Dislocation.ab,ti.
20. Diastasis.ab,ti.
21. Injury.ab,ti.
22. Rockwood.ab,ti.
23. 18 OR 19 OR 20 OR 21 OR 22
24. 8 AND 17 AND 23

## Registration

PROSPERO 2021 CRD42021291349.

## References

[1] Gorbaty JD, Hsu JE, Gee AO. Classifications in Brief: Rockwood Classification of Acromioclavicular Joint Separations. Clin Orthop Relat Res. 2017; 475(1): 283–287. DOI: 10.1007/s11999-016-5079-6 27637619 PMC5174051

[2] Moher D, et al. Preferred reporting items for systematic reviews and meta-analyses: the PRISMA statement. BMJ. 2009; 339: b2535. DOI: 10.1136/bmj.b2535 19622551 PMC2714657

[3] Shamseer L, et al. Preferred reporting items for systematic review and meta-analysis protocols (PRISMA-P) 2015: elaboration and explanation. BMJ. 2015; 350: g7647. DOI: 10.1136/bmj.g7647 25555855

[4] Higgins JPT, Thomas J, Chandler J, Cumpston M, Li T, Page MJ, Welch VA. (eds.). Cochrane Handbook for Systematic Reviews of Interventions version 6.2 (updated February 2021). Cochrane; 2021. Available from www.training.cochrane.org/handbook.

[5] Sterne JAC, et al. RoB 2: a revised tool for assessing risk of bias in randomised trials. BMJ. 2019; 366: l4898. DOI: 10.1136/bmj.l4898 31462531

[6] Jadad ARM, Carroll RA, Jenkinson D, Reynolds C, Gavaghan DJM, McQuay DJHJ. Assessing the Quality of Reports of Randomized Clinical Trials: Is Blinding Necessary? Controlled Clin Trials. 1996; 17: 1–12. DOI: 10.1016/0197-2456(95)00134-4 8721797

[7] Balshem H, et al. GRADE guidelines: 3. Rating the quality of evidence. J Clin Epidemiol. 2011; 64(4): 401–6. DOI: 10.1016/j.jclinepi.2010.07.015 21208779

[8] Guyatt GHO, Vist AD, Kunz GE, Falck-Ytter R, Alonso-Coello Y, Schunemann PHJ. GRADE: an emerging consensus on rating quality of evidence and strength of recommendations. BMJ. 2008; 336: 924–926. DOI: 10.1136/bmj.39489.470347.AD 18436948 PMC2335261

[9] Langer G, et al. GRADE guidelines: 1. Introduction – GRADE evidence profiles and summary of findings tables. Z Evid Fortbild Qual Gesundhwes. 2012; 106(5): 357–68. DOI: 10.1016/j.zefq.2012.05.017 22818160

